# Male genital injuries attending a tertiary hospital in a western region of Nepal: A two-year snapshot.

**DOI:** 10.12688/f1000research.52053.1

**Published:** 2021-05-04

**Authors:** Alok Atreya, Suman Baral, Ritesh G Menezes, Samata Nepal

**Affiliations:** 1 Lumbini Medical College, Palpa, Lumbini, 32500, Nepal; 2 College of Medicine, Imam Abdulrahman Bin Faisal University, Dammam, Saudi Arabia

**Keywords:** genital injuries, male, Nepal, penile fracture, scrotal laceration

## Abstract

Background: Male genital injuries are urological emergencies which if not promptly treated with correct therapeutic intervention may lead to chances of loss of fertility due to infections and anatomical disruption of normalcy. This study highlights the clinical scenarios, etiology and outcome of male genital injury cases that were managed at a tertiary care center in Nepal. Such injuries are not frequently encountered as lack of reporting by patients means cases are rare. The present study is the first from Nepal which depicts a comprehensive report on male genital injuries.

Methods: A retrospective analysis of discharge summaries was carried out and the cases of male genital injuries were reviewed during June 2020. All the treated cases during the two-year period from April 2018 to April 2020 at Lumbini Medical College, Nepal were included in the study.

Results: There were eight cases of genital trauma admitted and treated during the study period. All the patients were males and age ranged from six to 71 years with a mean age of 33 ± 21.45 years. Fall injury and road traffic accidents (RTA) were observed to be the primary cause in the majority of cases.

Conclusion: Superficial injuries to the penis and scrotum do not require surgical exploration and could be managed conservatively. However, deeper and complicated injuries, testicular preservation, the functionality of the part and cosmetic issues are taken into consideration which might require a multi-disciplinary approach. Apart from the medical issues pertaining to genital injuries, there are legal and psychological aspects of such events too which should not be ignored.

## Introduction

Genitals play a vital role in providing passage for metabolic waste along with procreation of new life. Protected within the groin they escape trauma; hence injuries are uncommon. However, when injured they are very painful and bleed profusely due to a rich vascular and nerve supply. Genital injuries are seldom life-threatening but may trigger psychological problems ^1, 2^.

There are few case reports from Nepal on male genital injuries, however, detailed studies describing the victim profile, nature and cause of injuries are a rarity. In this regard, the present research is a first from Nepal that attempts to study the cases of male genital trauma in detail to describe the profile of victims, circumstances of injury, modality of treatment and duration of hospital stay.

## Methods

A retrospective review of discharge summaries from the Department of Surgery of the Lumbini Medical College, Palpa, Nepal over the two-year period from April 2018 to April 2020 was conducted to identify patients treated for perineal injuries. The study included all the cases that had a history of injury to the male genitalia. The cases were identified by manual search based upon the diagnosis and history recorded. Discharge summaries with any of the keywords: penis, penile, scrotum, scrotal, perianal or perineum were selected. The case details were recorded for all the cases included in the study. Specific variables investigated in the study were age and gender of the patient, nature and manner of injury, mechanism of injury, anatomic site, treatment procedure, intra-operative findings and duration of hospital stay. The data thus obtained was then entered into a pre-structured proforma and exported to a Microsoft Excel 2018 spreadsheet and the results were expressed in frequencies, percentages, mean and standard deviation. The research was conducted in the Department of Forensic Medicine during June 2020 at Lumbini Medical College, Palpa, Nepal. The study was approved by the Institutional Review Committee of Lumbini Medical College, letter number IRC-LMC 08-D/020. Furthermore, permission to access and collect departmental data was obtained from the Professor and Head, Department of Surgery.

## Results

 A total of 15,125 cases were admitted during the study period in the Department of General Surgery of whom only eight were cases of genital trauma who were admitted and treated. All the victims were male and age ranged from six to 71 years with a mean age of 33 ± 21.45 years. Fall injury and road traffic accidents (RTA) were observed to be the primary cause for perineal trauma in the majority of cases. Table 1 depicts the characteristics of victims during the study period. Mean duration of hospital stay was 4.37 ± 1.41 days.

**Table 1.** Characteristics of male patients admitted and treated for genital trauma in the two years, April 2018 to April 2020 at Lumbini Medical College, Nepal (n=eight).

Age (years)	Diagnosis	As a consequence of	Management	Days of admission
6	Scrotal hematoma	Fall injury	Incision and drainage	6
17	Scrotal laceration	Perforation by snooker cues stick	Wound debridement and suturing	2
18	Scrotal hematoma	Road traffic accident	Aspiration of hematoma under intravenous anesthesia	5
24	Penile fracture	Traumatic sexual intercourse	Exploration and repair	5
31	Penile laceration	Fall Injury	Wound debridement (necrotic skin), circumcision and reconstruct	6
48	Scrotal hematoma	Road traffic accident	Aspiration of hematoma under intravenous anesthesia	4
49	Penile fracture	Masturbation	Exploration and repair	3
71	Scrotal laceration	Domestic violence	Wound debridement and suturing	4

## Discussion

Genital injuries in women are more common than men and mostly result from childbirth. Perineal tears in women related to childbirth are repaired by obstetricians. Forensic experts too encounter genital injuries in women during medicolegal evaluation of alleged sexual offences. In both circumstances the specific pattern of injury in women can help readily identify the nature of trauma. Genital injuries in women are usually easily repaired. On the other hand, there are diverse circumstances where the projected phallus in males could sustain traumatic injuries. As these traumas are rare, general surgeons may not be familiar with such cases due to a lack of specialist centers and on-site expertise in genito-urethral surgeries ^3^. Management of male genital trauma is always a challenge because of cosmetic and functional requirements.

The mechanism and severity of injury in male genitalia varies depending upon the location and the state of erectness or flaccidity ^4^. The flaccidity of the pendulous portion of the phallus has a tremendous capacity to resist trauma as it restricts transfer of kinetic energy ^4^. On the other hand, the root which is fixed, is more prone to trauma in straddle injury or pelvic fractures. Due to the increased pressure within, the penis is more vulnerable to injury when erect. The loose and lax genital skin in the males can deform and slide away from the point of contact, however, if injured there is the potential of avulsion stripping entire penile and scrotal skin ^4^.

The manner of injury in most cases was likely be accidents based upon the cause of injuries. Road traffic injuries and fall from height were the common reasons behind most cases of perineal injuries in the present study. Due to difficult geographical terrain in mountainous Nepal, most earthen and gravel roads are bumpy. Lack of maintenance and repair on the asphalt paved roads means they are also not free of potholes. Sudden jerk of the two wheelers (motorbikes and bicycles) in such potholes was the reason behind blunt scrotal trauma in one of the three victims of road traffic accidents in the present study. The discharge summary of the other victim revealed blunt scrotal trauma leading to hematoma formation due to a sudden jerk while driving a farm tractor. One patient was hit by a motorbike and sustained injuries to the scrotum.

In rural Nepal, the majority of people are engaged in agricultural activities. Degloving injury of the phallus with fracture was observed in one case, who had given a history of falling from a tree while collecting cattle fodder. Literature reveals grinding and crushing injuries to the scrotum while operating machinery, laceration following animal attack with or without complete amputation, burns, gunshot injuries to mention a few which were not reported in the present study ^1, 4– 6^.

Literature reveals that cultural politics from the mid-nineteenth century onwards considered sexuality a distinct entity separated from reproduction ^7^. This became apparent with a concern for the pleasure for women, sexual orientation, sexual rights and norms ^7^. It is argued that to become sexually aroused and experience orgasm is a normal physiological urge similar to eating, breathing or sleeping ^8^. Furthermore, human sexual response is believed to trigger reward centers associated with sex, both sexual or non-sexual in nature ^8^. Sex is not only a coitus for reproduction but it is a sensual pleasure too ^9, 10^. As an active agent in a sexual act, males consider the length of the penis to be associated with being macho and vigor. However, studies depict sexual pleasure as more associated with psychological and psychosocial factors rather than physical factors ^11^. Masturbation in men is considered a compensation for unavailable sex ^12^. Aggressive sexual acts were the reason behind penile fracture in two cases in the present study.

Although not life threatening, genital injuries in males result in excruciating pain. Furthermore, the location of the injury in the genital area makes the person feel embarrassed and, in the meantime, reluctant to seek medical help. This might be one of the reasons for the under reporting of genital injuries.

Male genital injuries range from simple swelling as a result of blunt trauma to more complicated injuries like avulsion and amputation. A multidisciplinary approach is required to manage a complicated male genital injury which involves a urosurgeon and a plastic surgeon. Psychological counselling is also an integral element in the treatment of such injuries. In cases where the restoration of the anatomical part is not possible in an amputated or a crushed phallus, the psychological trauma the victim will undergo would be more severe as he might not only feel embarrassment but also feel low about losing his masculinity. 

The head and neck of victims are the prime target for perpetrators in cases of violence and homicide. However, genitals are commonly involved in order to intimidate someone ^13, 14^. Sexual abuse is rarely reported as the victim is ashamed and embarrassed ^14^. This provides the perpetrator the courage and impunity for further heinous crimes ^14^. Although the intense pain in the genitalia might not last long the psychological trauma is grave which the victim has to bear throughout the rest of his life ^15, 16^. The dignity, self-respect and confidence of the person is shattered ^17^. The present study reported a case where a 71-year-old presented with scrotal laceration. The history was suggestive of domestic violence. In another case a 17-year-old sought medical attention after sustaining a penetrating injury of the scrotum with a snooker cue stick. Although the manner of injury sustained in this case is not mentioned, a possibility of sexual violence cannot be ignored. Patriarchal Nepalese society considers homosexual activity as a taboo; shame, guilt and fear of isolation constitute important reasons for not reporting such cases ^14^. 

In accordance with Article 192, Section 2, Subsection ‘F’ of the criminal code of Nepal ^18^, loss of masculinity as a consequence of physical assault is prosecuted under grievous injury and the perpetrator can be given a custodial sentence with 10 years of imprisonment and a fine of one lakh rupees.

Proper functioning of sexual organs is important not only for reproduction but for married life too. Male impotency is a ground for divorce in Nepal ^19^. Loss of sexual function due to traumatic injury to the spinal cord or associated nerves as a consequence of accident or intentional trauma is liable for compensation or a fine. 

The retrospective nature of this study has its own inherent limitations. We could not find any previous studies from Nepal to compare the findings of the present study. Long term follow-up of the patients to study late complications in the form of urethral stricture, penile curvature, fertility and sexual functions could not be examined.

## Conclusion

Genital injuries, although a rarity in south east Asian medical literature, are commonly encountered in south east Asian countries including Nepal. Although most of the injuries sustained are accidental in nature, there are instances where the nature of injuries is intentional which could be elucidated by a detailed history. Anatomical repair of the trauma along with psychosocial and legal aspects of the event are not to be ignored. A multidisciplinary team approach is often required in the management of a case of male genital injury which should consist of a general surgeon, urosurgeon, plastic surgeon, psychiatrist and a medicolegal expert.

## Data availability

All data underlying the results are available as part of the article and no additional source data are required.

## Consent

Consent for publication from patients or their families is not applicable to the present study because the present study is record-based where retrospective review of discharge summaries was done. The Institutional Review Committee of Lumbini Medical College, letter number IRC-LMC 08-D/020 which approved the present study agreed to waive the patient consent in this instance. The anonymity of the patient was maintained and the data were anonymized, however, these alterations did not distort the scientific data.
