# Supplementary material for: Male genital injuries treated at a tertiary hospital in the western region of Nepal: A two-year snapshot
Source: F1000Res. 2022 Jan 6;10:337. Originally published 2021 May 4. [Version 2] doi: 10.12688/f1000research.52053.2 (PMC8609399; doi:10.12688/f1000research.52053.2)
Supplement: Peer review history

# Peer review: Reviewer response for version 1

**DOI:** 10.5256/f1000research.55279.r100604

**Published:** 2021-11-22

**Authors:** Raghvendra Singh Shekhawat

**Affiliations:** 1 Department of Forensic Medicine and Toxicology, All India Institute of Medical Sciences, Jodhpur, Jodhpur, Rajasthan, India

These findings have promise for urology emergency clinical care. There should be more information on female genital damage, especially in relation to sexual abuse. The socio-cultural context of genital injuries is well-described by the authors. The authors have also managed to touch on the forensic aspects of the matter by mentioning the appropriate sections of the Nepalese penal code. Longer-term repercussions of such injuries in terms of physical and psychological well-being may be included in the discussion section. While the authors' efforts are praiseworthy, additional research with a larger sample size is suggested.

Is the work clearly and accurately presented and does it cite the current literature?

Yes

If applicable, is the statistical analysis and its interpretation appropriate?

Yes

Are all the source data underlying the results available to ensure full reproducibility?

Yes

Is the study design appropriate and is the work technically sound?

Yes

Are the conclusions drawn adequately supported by the results?

Yes

Are sufficient details of methods and analysis provided to allow replication by others?

Yes

Reviewer Expertise:

Forensic Pathology

I confirm that I have read this submission and believe that I have an appropriate level of expertise to confirm that it is of an acceptable scientific standard.

# Response

**Published:** 2021-11-23

**Authors:** Samata Nepal

**Affiliations:** Lumbini Medical College, Nepal

Thank you for your time reviewing our manuscript and for your positive feedback. As the manuscript focused upon all the cases of male genital injuries treated at Lumbini Medical College, the study did not include genital injuries in females. As this was a prevalence study we did include all the cases during the two-year period (2018- 2020). Although genital injuries are not uncommon, most of the cases go to the specialty hospital with an uro-surgery facility, which is one of the reasons for the small sample size in the present study. We value your suggestion and would conduct future studies with a larger time frame and large sample.

# Peer review: Reviewer response for version 1

**DOI:** 10.5256/f1000research.55279.r84442

**Published:** 2021-06-10

**Authors:** Ramkaji Baniya

**Affiliations:** 1 Our Lady of Lake Our Lady of the Lake Regional Medical CenterMedical Center, Baton Rouge, LA, USA, Baton Rouge, LA, USA

**Minor changes:**

 Abstract: 

1. First paragraph second line: Delete "chances of".
2. Fourth paragraph. Please rephrase the sentence as "Such injuries are not frequently encountered as patient are hesitant to report such injuries." 
3. Second paragraph first line: Delete "was carried out and " and replace with "of".
4. Mention how the cases were managed briefly in the result section on the abstract and the manuscript. 

Introduction: 

1. First paragraph: replace "genitals play" with "Human genitalia plays". 
2. Second paragraph: replace "victim" with "patient". 
3. Second paragraph: replace "are a rarity" with "have not been done". 

Discussion: 

1. First paragraph, sixth sentence rephrase as "As these traumas are rare, general surgeons may not be familiar with these urosurgical repair."
2. Third paragraph first sentence. Rephrase a "the manner of injury in most cares were accidents." 
3. Third paragraph. Please rephrase the third, fourth and fifth sentences as " Due to difficult geographical landscape and poorly maintained roads, road traffic accidents are common in Nepal. Sudden deceleration of a two wheeler on a pothole was the cause of blunt scrotal trauma  in one of the three victims of road traffic accidents in the present study."
4. Fourth paragraph, second sentence. Change "falling" to "fall". Delete "while collecting cattle fodder."
5. I would recommend deleting the fifth paragraph. The last sentence of the paragraph can be added to the fourth paragraph.
6. Seventh paragraph, last sentence rephrase as "In cases where the restoration of the anatomical part is not possible in an amputated or a crushed phallus, the psychological trauma to the patient is enormous. Patients are likely to feel embarrassed and sexually incompetent." Cite PMID **24560873** ^1^

Is the work clearly and accurately presented and does it cite the current literature?

Yes

If applicable, is the statistical analysis and its interpretation appropriate?

Yes

Are all the source data underlying the results available to ensure full reproducibility?

Yes

Is the study design appropriate and is the work technically sound?

Yes

Are the conclusions drawn adequately supported by the results?

Yes

Are sufficient details of methods and analysis provided to allow replication by others?

Yes

Reviewer Expertise:

Meta-analysis

I confirm that I have read this submission and believe that I have an appropriate level of expertise to confirm that it is of an acceptable scientific standard.

## References

[1] : The impact of genital trauma on wounded servicemen: qualitative study. *Injury* .2014;45(5) : 10.1016/j.injury.2013.12.009 825-9 10.1016/j.injury.2013.12.009 24560873

# Response

**Published:** 2021-11-23

**Authors:** Samata Nepal

**Affiliations:** Lumbini Medical College, Nepal

Thank you for your time reviewing our manuscript and for your positive feedback. All the changes and edits that you have suggested are incorporated in the revised version.
